# Transcriptomic and anatomical complexity of primary, seminal, and crown roots highlight root type-specific functional diversity in maize (*Zea mays* L.)

**DOI:** 10.1093/jxb/erv513

**Published:** 2015-11-30

**Authors:** Huanhuan Tai, Xin Lu, Nina Opitz, Caroline Marcon, Anja Paschold, Andrew Lithio, Dan Nettleton, Frank Hochholdinger

**Affiliations:** ^1^Institute of Crop Science and Resource Conservation, Crop Functional Genomics, University of Bonn, D-53113 Bonn, Germany; ^2^Experimental Medicine and Therapy Research, University of Regensburg, D-93053 Regensburg, Germany; ^3^Department of Statistics, Iowa State University, Ames, IA 50011-1210, USA

**Keywords:** Crown root, primary root, RNA-seq, root type, seminal root, transcriptome, *Zea mays*.

## Abstract

Maize develops a complex root system composed of embryonic and post-embryonic roots. Spatio-temporal differences in the formation of these root types imply specific functions during maize development. A comparative transcriptomic study of embryonic primary and seminal, and post-embryonic crown roots of the maize inbred line B73 by RNA sequencing along with anatomical studies were conducted early in development. Seminal roots displayed unique anatomical features, whereas the organization of primary and crown roots was similar. For instance, seminal roots displayed fewer cortical cell files and their stele contained more meta-xylem vessels. Global expression profiling revealed diverse patterns of gene activity across all root types and highlighted the unique transcriptome of seminal roots. While functions in cell remodeling and cell wall formation were prominent in primary and crown roots, stress-related genes and transcriptional regulators were over-represented in seminal roots, suggesting functional specialization of the different root types. Dynamic expression of lignin biosynthesis genes and histochemical staining suggested diversification of cell wall lignification among the three root types. Our findings highlight a cost-efficient anatomical structure and a unique expression profile of seminal roots of the maize inbred line B73 different from primary and crown roots.

## Highlight

Transcriptomic and anatomical analyses of primary, seminal, and crown roots of the maize inbred line B73 revealed root type-specific diversity related to the different functions of these root types during development.

## Introduction

Roots play a vital role in plant development and fitness because they provide mechanical support, mediate water and nutrient uptake from soil, and interact with microbial communities in the rhizosphere (reviewed in Hawkes *et al.*, 2007; Zhu *et al.*, 2011; Villordon *et al.*, 2014). Sophisticated root system architecture is a prerequisite for optimal capturing of soil resources (Giehl and von Wirén, 2014). For instance, in maize, a deeper root system enhances drought tolerance (Ribaut *et al.*, 2009), while shallower root angles of seminal and crown roots increase phosphorus acquisition (Zhu *et al.*, 2005).

The root stock of maize consists of an embryonically formed primary root and a variable number of embryonic seminal roots and post-embryonic shoot-borne crown and brace roots (reviewed in Feldman, 1994; Hochholdinger *et al.*, 2004a, b). These root types are initiated from distinct tissues during successive stages of development. Primordia of primary roots are formed at the basal pole of the embryo and become visible as distinct structures within the embryo ~10–15 d after pollination (Tillich, 1977). After germination, the primary root emerges within 2–3 d. Seminal root primordia are formed in the embryo 22–40 d after pollination (Erdelska and Vidovencova, 1993). The number of seminal roots is largely determined by their genetic background (Sass, 1977; Erdelska and Vidovencova, 1993). Seminal roots are initiated at the scutellar node ~1 week after germination. Up to 12 seminal roots are formed per seedling, which thus dominate the seedling root stock (Abbe and Stein, 1954). In the first weeks after germination, primary and seminal roots make up the major portion of the seedling root stock (Hochholdinger *et al.*, 2004b) and are thus vital for the early vigor of young maize seedlings (Peter *et al.*, 2009). In contrast, the post-embryonic shoot-borne root system shapes the adult root stock (Martin and Harris, 1976). Shoot-borne roots formed from consecutive shoot nodes below the soil level are designated crown roots, while brace roots are initiated from above-ground shoot nodes (Hochholdinger *et al.*, 2004a). The first whorl of shoot-borne crown roots is formed ~10–14 days after germination (dag) (Hochholdinger *et al.*, 2004b). An adult maize root stock develops ~70 shoot-borne roots, which are organized on average in six whorls of underground crown roots and 2–3 whorls of above-ground brace roots (Hoppe *et al.*, 1986). A commonality of all major embryonic and post-embryonic root types is their ability to initiate post-embryonic lateral roots from pericycle and endodermis cells (Fahn, 1990).

A number of maize developmental mutants with specific defects in their root system architecture have been identified. These mutants are affected in shoot-borne root formation (Hetz *et al.*, 1996), lateral root formation (Hochholdinger and Feix, 1998; Hochholdinger *et al.*, 2001; Woll *et al.*, 2005), or root hair formation (Wen and Schnable, 1994; Wen *et al.*, 2005; Hochholdinger *et al.*, 2008; Nestler *et al.*, 2014). Among those, seminal and shoot-borne root formation are controlled by *rtcs* (*rootless concerning crown and seminal roots*) (Hetz *et al.*, 1996) which encodes an auxin-inducible LOB (lateral organ boundaries) domain transcription factor (TF) (Taramino *et al.*, 2007) acting downstream of the auxin response factor ARF34 (Majer *et al.*, 2012; Xu *et al.*, 2015). The *rum1* (*rootless with undetectable meristems 1*) gene encodes a monocot-specific Aux/IAA protein (von Behrens *et al.*, 2011) that controls the formation of seminal roots and lateral roots at the primary root (Woll *et al.*, 2005). These genetic studies supported the notion of root type-specific control of maize root system formation (Hochholdinger *et al.*, 2004a). Gene expression profiles of different maize root tissues generated by microarray analyses (Woll *et al.*, 2005; Jiang *et al.*, 2006; Dembinsky *et al.*, 2007; Muthreich *et al.*, 2013) and RNA sequencing (RNA-seq) studies (Paschold et al., 2012, 2014; Zhang *et al.*, 2014) provided initial cues of molecular networks involved in maize root development. In this study, we applied RNA-seq to profile global expression patterns of genes expressed in primary, seminal, and crown roots of the maize inbred line B73 during early development. In parallel, a detailed comparative investigation of anatomical and histochemical features across the root types was conducted. The goal of this study was to survey anatomical and transcriptomic differences to reveal functional differences between the different root types.

## Materials and methods

### Plant material and growth conditions

Seeds of the maize inbred line B73 were surface sterilized with 6% hypochlorite for 10min, and then germinated in paper rolls imbibed in distilled water for 2 d (Hetz *et al.*, 1996). Subsequently, these paper rolls were transferred to Hoagland’s solution (Zhao *et al.*, 2012) in a growth chamber (16h light, 28 °C, and 8h dark, 22 °C; 60% humidity) until the roots were harvested. The culture solution was replaced every second day.

### RNA sequencing and read mapping

For RNA-seq, pools of 10 roots (length: 20–30mm) per root type were sampled per biological replicate. For each root type, four independent biological replicates were analyzed. The sampled plant material was immediately frozen in liquid nitrogen and stored at –80 °C until RNA isolation.

Total RNA was extracted by using the RNeasy Plant Mini Kit (Qiagen, Venlo, The Netherlands). RNA integrity and quality were assayed by agarose gel electrophoresis and a Bioanalyzer using an Agilent RNA 6000 Nano Chip (Agilent Technologies, Santa Clara, CA, USA). High quality samples with RIN (RNA integrity number) values >8 were used for RNA-seq. cDNA libraries were prepared according to the manufacturer′s protocol (Illumina, San Diego, CA, USA) and subsequently sequenced on an Illumina HiSeq 2000 platform to generate 90bp paired-end reads. Alignment of the processed raw reads to the maize reference genome sequence version 2 (B73 RefGen_v2) was performed with the CLC Genomics Workbench (Version 7.0.1; http://www.clcbio.com/products/clc-genomics-workbench/). Stacked reads that shared the same starting point, sequence, and orientation were merged into one read. Maximum gaps of 50kb were allowed to span introns. A read was considered only if at least 75% of its sequence fitted with 90% similarity to the reference sequence. Mapped reads were further projected to the filtered gene set (FGS v2; Release 5b, ftp://ftp.gramene.org/pub/gramene/maizesequence.org/release-5b/filtered-set/). Only reads that mapped uniquely to the filtered gene set with 80% of their length displaying 90% similarity were considered in subsequent analyses. RNA-seq data have been deposited in the NCBI sequencing read archive (SRA; http://www.ncbi.nlm.nih.gov/sra; Accession: SRP052697).

### Statistical analyses to determine active and differentially expressed genes

The activity status of genes (on/off) was determined by fitting a hierarchical model to read count data. Read counts were modeled as draws from negative binomial distributions where the variance is a quadratic function of the mean. The negative binomial distributions were parameterized with a mean µ, dispersion parameter φ, and variance µ+φµ2. To account for the experimental design, randomly distributed lane effects were included in the model. Conditional on random effects, each gene was considered as a draw from a negative binomial distribution with a gene-specific dispersion parameter and a mean determined by the sum of a normalization factor and a linear combination of gene-specific fixed and random effects. The normalization factor for each sample and gene was taken as the fitted value of a sample-specific smoother using the log of the raw read count plus one as the response, and GC content and log of gene length as covariates. The log of the dispersion parameter and each fixed effect were considered as independent draws from separate normal distributions, and the precisions for the random effects were modeled as draws from gamma distributions. The parameters of each normal distribution and the distribution corresponding to the lane random effects were estimated through an empirical Bayes procedure. Activity or inactivity of a gene was determined by computing the posterior probability that the expected number of reads is less than a given threshold. A gene was declared as ‘inactive’ in a specific root type if the resulting posterior probability was >0.5. The R-package ShrinkBayes, which is based on the ideas of Van de Wiel *et al.* (2012), was used to obtain empirical Bayes estimates of hyperparameters and compute the desired posterior probabilities. A key idea of ShrinkBayes is to use Integrated Nested Laplace Approximations (INLAs) to obtain very fast, but accurate, approximations of the marginal posteriors.

For differential expression analysis, gene expression was normalized as FPKM (fragments per kilobase of exon model per million mapped reads) values. The R-package limma based on linear models (Ritchie *et al.*, 2015) was used for determination of differentially expressed genes in pairwise comparisons. Euclidean algorithm based K-means clustering [false discovery rate (FDR) <5%] was performed, to generate 12 differential expression patterns among the three root types.

### PCA and cluster analysis

A PCA (principal component analysis) was performed by using the prcomp function in R with default settings. Hierarchical clustering of samples was performed based on Pearson correlations in the CLC Genomics Workbench. Log_2_-transformed FPKM values were used for both PCA and hierarchical clustering.

Heat maps of expression levels of differentially expressed TFs and lignin biosynthesis genes were based on log_2_-transformed FPKM values and were generated via combined tools of Gene Cluster 3.0 based on Euclidean distances (de Hoon *et al.*, 2004) and the Java Tree View (Saldanha, 2004).

### Functional annotation of differently expressed genes

Gene Ontology (GO) analyses were conducted using singular enrichment analysis (SEA) with an online agriGO platform (http://bioinfo.cau.edu.cn/agriGO/analysis.php; Du *et al.*, 2010). SEA compares input list of genes (the number of root type exclusively expressed genes or differentially expressed genes in this study) with reference genes (24 687 expressed genes) to identify enriched GO terms. Resulting *P*-values were further adjusted by introducing FDR <5% (Benjamini and Yekutieli, 2001).

Significantly over- and under-represented categories were based on the MapMan annotation (http://mapman.gabipd.org/web/guest/mapman; Thimm *et al.*, 2004). Declaration of a significantly changed category was based on comparison of detected genes with expected number in the category (χ^2^ tests with Yate’s continuity correction). The expected number of genes per category was calculated based on the distribution of all expressed genes in the MapMan categories.

### Anatomy and histochemistry

Fresh hand cross-sections of 30, 60, and 90mm long roots were prepared for each root type in 10mm increments for anatomical analyses. Segments of samples were fixed in 4% paraformaldehyde in 1× phosphate buffer (containing 10mM NaH_2_PO_4_ and 10mM Na_2_HPO_4_, pH 7) and then sectioned by hand. Transverse sections were stained with 0.1% toluidine blue for 30s, washed, and mounted in 50% xylene, and then photographed under a bright field microscope (PixCell IIe System, Zeiss, Jena, Germany). ImageJ2 software (version 2.0.0-rc-28; http://fiji.sc/Downloads) was used for the quantification of anatomical traits. Cell wall lignification was visualized by berberine–aniline blue staining according to Brundrett *et al.* (1988). After staining, fluorescence was observed under a PixCell IIe microscope (Zeiss) using the excitation filter G365, the chromatic splitter FT395, and the barrier filter LP 420.

## Results

### Morphological and anatomical characterization of maize primary, seminal, and crown roots early in development

**Fig. 1.**
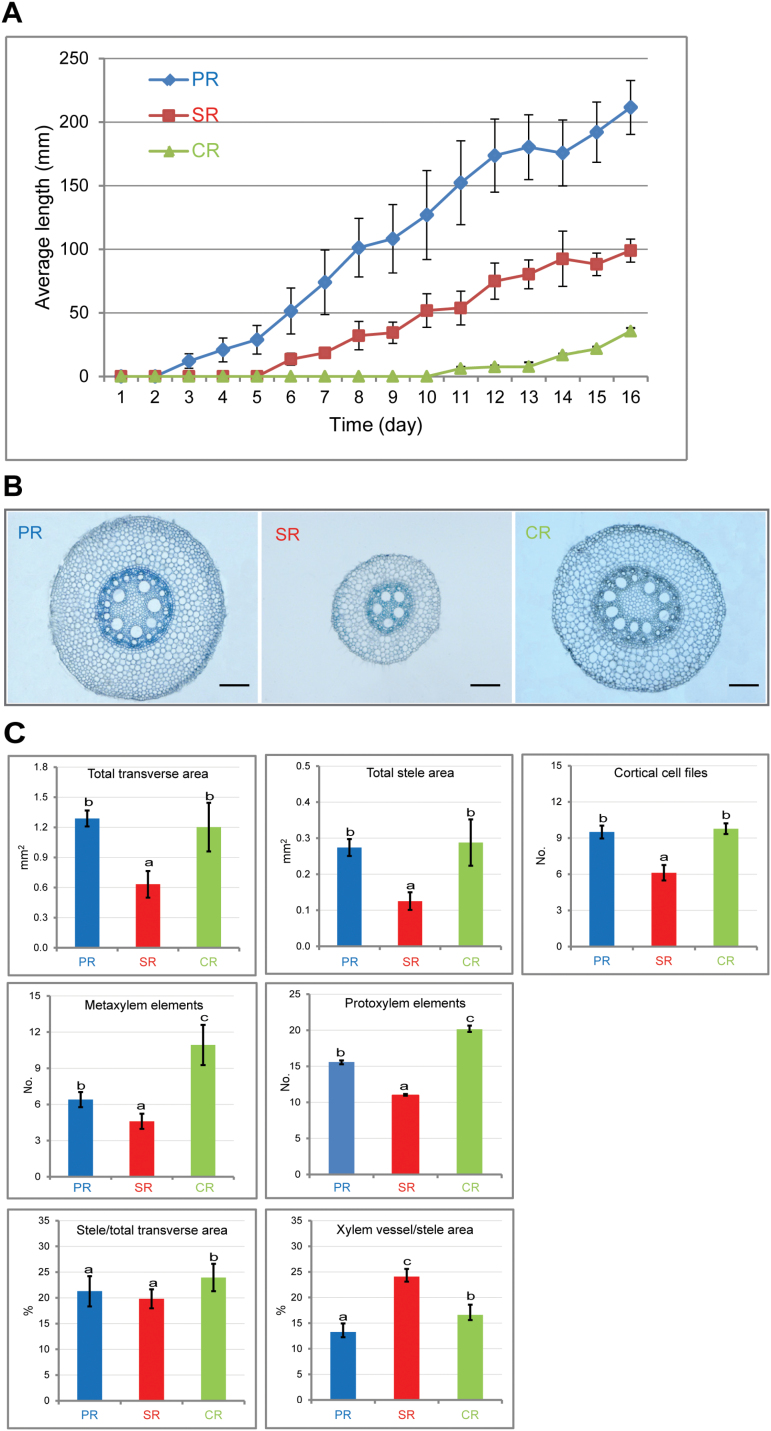
Morphological and anatomical analyses of maize primary, seminal, and crown roots. (A) Growth of the three root types within 16 d after germination (*n*=30 per time point; error bars indicate ±SD). (B) Transverse sections of the proximal parts of 20mm long roots of each type stained with toluidine blue. Scale bars=200 μm. (C) Quantification of root anatomical traits in transverse sections of the three root types (*P*<0.05, *n*=15; error bars indicate ±SD). PR, primary roots; SR, seminal roots; CR, crown roots.

In maize, different root types are formed consecutively in early development. In the inbred line B73, the primary root emerges 2–3 dag, seminal roots become visible 5–6 dag, and shoot-borne crown roots are formed between 10 and 11 dag (Fig. 1A). The number of seminal roots is variable between different individuals of a genotype. In the inbred line B73, the number of seminal roots varies between two and four (Supplementary Table S1 available at *JXB* online). Moreover, the number of seminal roots varies widely between different genotypes. In a panel of 30 different maize inbred lines, the inbred line UH005 did not form any seminal roots while the inbred line CML322 formed on average 5.4 seminal roots (Supplementary Table S1). Individuals of the inbred line Ki11 formed up to 10 seminal roots (Supplementary Table S1).

To study the anatomical structure of B73 primary, seminal, and crown roots, serial transverse sections were compared in a developmental time course experiment in roots of 30, 60, and 90mm length in 10mm increments (Supplementary Fig. S1 at *JXB* online). Within each of the three root types, no difference in their anatomical organization was observed along the longitudinal axes and between the developmental stages (Supplementary Fig. S1). Subsequently, transverse sections of the proximal parts of B73 primary, seminal, and crown roots of 20–30mm length were subjected to a detailed comparative anatomical analysis (Fig. 1B). These microscopic analyses revealed that seminal roots of the inbred line B73 have a smaller diameter and a different organization of the vasculature compared with primary and crown roots. Quantification of anatomical parameters (Fig. 1C) in transverse orientation demonstrated that embryonic seminal roots displayed a significantly smaller total area, a smaller total stele area, and a reduced number of cortical cell files compared with primary and crown roots (Fig. 1C). Crown roots displayed the highest number of meta- and protoxylem elements, followed by primary and seminal roots (Fig. 1C). Finally, the proportion of stele area relative to the total root area was not different in primary and seminal roots, while it was slightly but significantly higher in crown roots. In contrast, the proportion of xylem vessels relative to the total stele area was significantly higher in seminal roots than in primary and crown roots (Fig. 1C).

### RNA-seq of maize primary, seminal, and crown root transcriptomes

To survey how their anatomical differences are reflected in global gene expression profiles, RNA-seq of B73 primary, seminal, and crown roots of 20–30mm length was performed in four biological replicates per root type. The RNA-seq experiment resulted in 22–24 million high quality paired-end 90bp reads per biological replicate (Supplementary Table S2 at *JXB* online). Among all reads, 82–87% mapped uniquely to the maize reference genome sequence (ZmB73_RefGen_v2; Supplementary Table S2). After removal of redundant reads sharing the same start and end co-ordinate, sequencing direction, and sequence (‘stacked reads’), between 81% and 87% of the remaining reads mapped uniquely to the ‘filtered gene set’ֹ, a set of 39 656 gene models predicted with high confidence (ZmB73 FGS_5B_FGSv2; Supplementary Table S2).

**Fig. 2.**
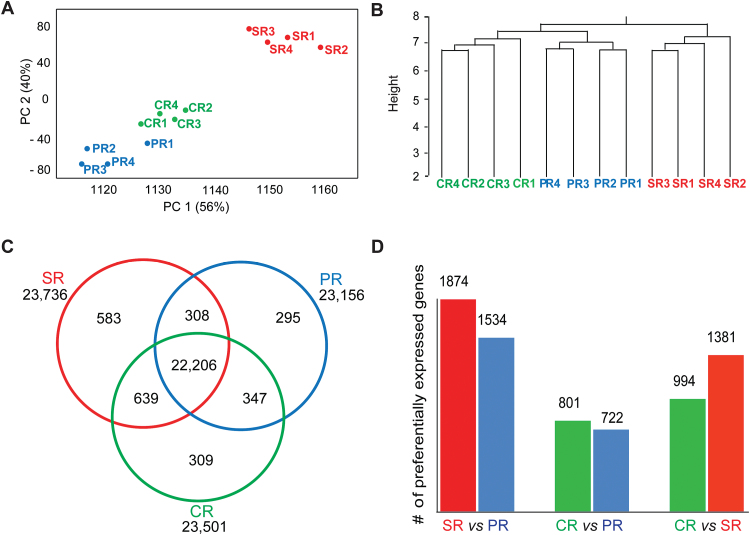
Relationship of root transcriptome samples, root type-specific gene expression, and differential gene expression. (A) Principal component analysis (PCA) of the 12 RNA-seq samples of the three root types. The first and second principal components collectively explain 96% of variance. (B) Hierarchical clustering of the 12 RNA-seq samples of the three root types based on Euclidian distance. The *y*-axis indicates the degree of the variance. (C) Number of genes with conserved and root type-specific expression in the three root types. (D) Pairwise comparisons of differentially expressed (FDR <5%, |log_2_Fc| ≥1) genes among the three root types. PR, primary roots; SR, seminal roots; CR, crown roots; PC 1 and PC2, principal component one and two.

To assess the reproducibility of the data set, relationships between the 12 samples were determined by a PCA (Fig. 2A) and hierarchical clustering (Fig. 2B). Both analyses showed high correlation among biological replicates of each root type. Furthermore, the transcriptomes of primary and crown roots clustered in close proximity, while a distinct cluster comprising the replicate samples of seminal roots was observed (Fig. 2A, B).

### Determination of the activity status of genes and their root type-specific expression

A hierarchical model with a negative binomial response (see the Materials and methods) was applied to determine the activity status (on/off) of genes in the three root types. In total, 24 687 of 39 656 (62%) high confidence gene models (ZmB73 FGS_5B_FGSv2) were expressed in at least one root type (Fig. 2C; Supplementary Table S3 at *JXB* online). Among those, 23 156 genes were expressed in primary roots, 23 736 in seminal roots, and 23 501 in crown roots (Fig. 2C). In total, 96% (22 206/23 156) of genes expressed in primary roots, 94% (22 206/23 736) of genes active in seminal roots, and 95% (22 206/23 501) of genes transcribed in crown roots were also active in the other two root types. In contrast, 1187 genes were exclusively expressed in only one of the three root types, comprising 583 seminal root-, 309 crown root-, and 295 primary root-specific genes (Fig. 2C). A GO analysis was conducted to identify enriched biological functions among these root type-specific genes. Enriched GO terms were calculated by SEA with agriGO (see the Materials and methods). Among seminal root-specific genes, only the GO term ‘transcription factor activity’ (GO: 0003 700) was enriched. Among crown root-specific genes, only the GO term ‘photosynthesis’ (GO: 0015 979) was over-represented. Among the primary root-specific genes, no GO term was enriched.

### Genes differentially expressed between distinct root types

Pairwise contrasts between the three root types were determined to identify differentially expressed genes. The distribution of fold change (Fc) versus mean relative expression revealed that differential expression was observed for genes with both low and high expression (Supplementary Fig. S2A at *JXB* online). In pairwise comparisons of the three root types, more differentially expressed genes (FDR <5%, |log_2_Fc| ≥1) were observed in comparisons involving seminal roots compared with contrasts determined from primary and crown roots (Fig. 2D; Supplementary Fig. S2B). In comparisons of seminal versus primary and crown roots, substantially more differentially expressed genes were preferentially expressed in seminal roots. SEA with agriGO (see the Materials and methods) revealed that molecular functions such as antioxidant activity, catalytic activity, and electron carrier activity were enriched among the differentially expressed genes in all three pairwise comparisons (Supplementary Fig. S3A–C). In addition, regulation of biological process, biological regulation, and transcriptional regulator activity were enriched in the comparisons of primary versus seminal roots and crown versus seminal roots (Supplementary Fig. S3A, C). Photosynthesis was enriched in the comparisons of primary versus crown roots and crown versus seminal roots (Supplementary Fig. S3B, C).

### Exploration of global gene expression patterns in the three root types

**Fig. 3.**
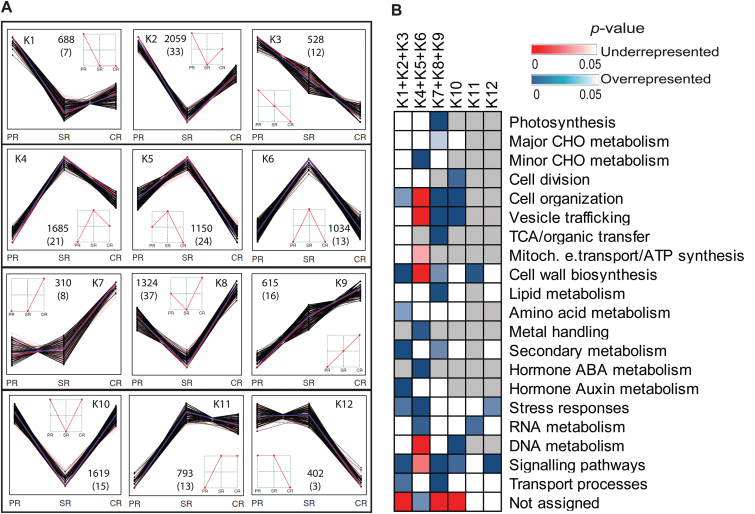
Global expression profiling of genes among the three root types. (A) K-means clustering showing all 12 possible expression patterns of 12 207 differentially expressed genes (202 classical maize genes in parentheses). Each black line represents a gene; the red lines represent median values; the blue lines represent the mean value. (B) Over- or under-representation of MapMan functional categories among six patterns representing peak expression in one or two root types. Clusters K1–K3 (primary roots), K4–K6 (seminal roots), and K7– K9 (crown roots) were combined in this analysis because they displayed peak expression in one of the three root types (χ^2^ tests, *P*<0.05; red, significantly under-represented; blue, significantly over-represented; white, not significant; gray, not detected). PR, primary roots; SR, seminal roots; CR, crown roots.

To explore differential expression across the three root types, K-means clustering (see the Materials ad methods) identified 12 clusters representing all possible patterns of differential expression (Fig. 3A; Supplementary Table S4 at *JXB* online). In total, 12 207 of 24 687 (49%) genes were classified into the 12 clusters, while the remaining genes (51%) were constitutively expressed. The 12 clusters were further classified into six patterns according to peak expression in one or two root types (Fig. 3B). Patterns K1–K3 displayed peak expression in primary roots, while patterns K4–K6 peaked in seminal roots and K7–K9 in crown roots (see Fig. 3A). In contrast, patterns K10, K11, and K12 displayed peak expression in two of three root types (see Fig. 3A). According to this classification, genes of pattern K4–K6 (3869/12 207, 32%) that displayed peak expression in seminal roots comprised the highest number of genes of the six patterns.

Over- and under-representation of MapMan functional categories was determined in the six patterns of differentially expressed genes compared with all expressed genes (Fig. 3B; see the Materials and methods). The analysis revealed that enriched or under-represented categories among genes preferentially expressed in primary (K1+K2+K3) or crown roots (K7+K8+K9) were relatively similar, whereas genes preferentially expressed in seminal roots (K4+K5+K6) displayed distinct enrichment patterns. For instance, functional categories such as cell organization, cell wall biosynthesis, secondary metabolism, signaling pathways, and transport processes were over-represented among genes preferentially expressed in primary and crown roots, while they were under-represented or not enriched among genes peaking in seminal roots. Genes involved in auxin metabolism such as *aux/iaa33* and *arf34* were enriched in primary roots. In contrast, genes that are required for minor CHO metabolism (such as sugar and sugar derivate metabolism), metal handling, abscisic acid (ABA) metabolism (including the key ABA biosynthesis genes *vp14* and *zep2* and numerous ABA-induced genes), and RNA metabolism were highly over-represented among genes with peak expression in seminal roots. These categories were under-represented or not significantly changed among genes preferentially expressed in primary and crown roots. Moreover, stress-related genes were highly over-represented in the group of genes peaking in seminal roots, and moderately enriched among genes peaking in primary roots. Genes required for cell wall biosynthesis and RNA metabolism were enriched in cluster K11, the group of genes preferentially expressed in both crown and seminal roots. Genes preferentially expressed in both primary and seminal roots (K12) were enriched in the functional categories stress and signaling.

### Preferential expression of classical maize genes in different root types

In maize, a set of 468 hand-curated ‘classical genes’ with experimentally confirmed function is available (Schnable and Freeling, 2011). In total, 348 of these classical genes were detected in this study. Among those, 202 were differentially expressed among the three root types and assigned to the 12 expression clusters defined in Fig. 3A. Novel root type-specific functions were revealed for classical maize genes displaying peak expression in one of the three root types (Supplementary Table S5 at *JXB* online) including 52 in primary roots (K1+K2+K3), 58 in seminal roots (K4+K5+K6), and 61 in crown roots (K7+K8+K9). For instance, among the group of 52 classical genes with peak expression in the primary root, seven of eight cellulose synthesis genes of the classical gene set were observed. Moreover, six of nine tubulin synthesis genes of the classical gene set were detected among the 61 genes displaying peak expression in crown roots. Finally, among 58 classic genes peaking in seminal roots, seven of 10 genes encoding aquaporin proteins and all four genes of the classical gene set translating into heat shock proteins were present. Furthermore, genes encoding stress-related proteins such as a WOUND INDUCED PROTEIN 1 (WIP1), an NaCl STRESS PROTEIN (NCA1), and a MAIZE INSECT RESISTANCE PROTEIN (MIR1) were highly expressed in seminal roots. The 31 classical genes assigned to clusters K10–K12 displayed diverse functions (Supplementary Table S5).

### Expression dynamics of transcriptional regulators in the three root types

**Fig. 4.**
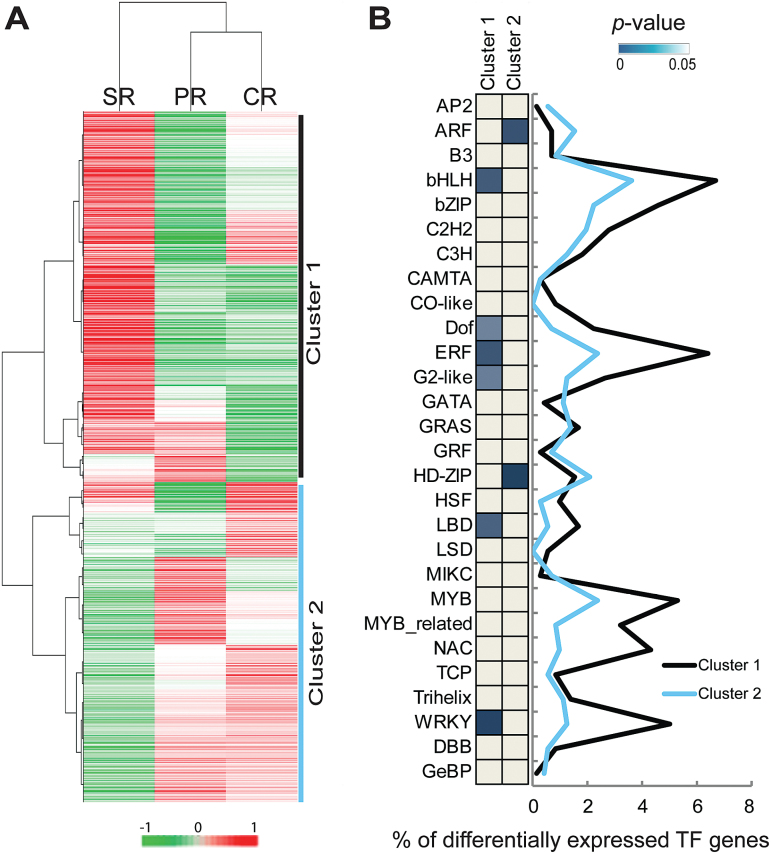
Dynamics of the expression profiles of transcriptional regulators. (A) Hierarchical clustering analysis of 1252 differentially expressed genes involved in transcriptional regulation of the three root types based on average expression values. (B) Classification of genes in 28 major transcription factor families of the two clusters from (A) as a proportion (in %) of all differentially expressed transcription factor genes. Significantly enriched families in each cluster were determined by Fisher’s exact tests (*P*<0.05) and indicated by blue blocks. Gray, not significant. PR, primary roots; SR, seminal roots; CR, crown roots.

Expression dynamics of genes involved in transcriptional regulation were surveyed to investigate their role in the developmental control of specific root types. In total, 2442 genes related to transcriptional regulation were identified based on their MapMan annotation. Among those, 1252 genes were differentially expressed between the three root types (Supplementary Table S6 at *JXB* online). Hierarchical clustering classified these genes into two clusters (Fig. 4A). Cluster 1, containing 684 (55%) of the differentially expressed genes, displayed high expression in seminal roots. The remaining 568 (45%) genes that were grouped into cluster 2 showed high expression in primary and crown roots (Fig. 4A). A comparison of these 1252 genes with the maize TF database PlantTFDB v3.0 (http://planttfdb.cbi.pku.edu.cn/; Jin *et al.*, 2013) identified 718 differentially expressed genes in the two clusters which encode TFs (457 in cluster 1 and 261 in cluster 2). Furthermore, family-specific expression trends of each cluster were determined by Fisher’s exact test (Fig. 4B). Six gene families encoding TFs [BASIC/HELIX–LOOP–HELIX (bHLH), DNA BINDING WITH ONE FINGER (DOF), ETHYLENE RESPONSE FACTOR (ERF), LATERAL ORGAN BOUNDARY DOMAIN (LBD), GOLDEN2 (G2)-like, and WRKY] were over-represented among genes with peak expression in seminal roots (cluster 1; Fig. 4B). In contrast, genes encoding the AUXIN RESPONSE FACTORs (ARFs) and the HOMEODOMAIN-LEUCINE ZIPPERs (HD-ZIPs) were over-represented in primary and crown roots (cluster 2; Fig. 4B). These results suggest distinct roles for transcriptional regulators in seminal root development compared with primary and crown roots.

### Root type diversification of cell wall lignification

**Fig. 5.**
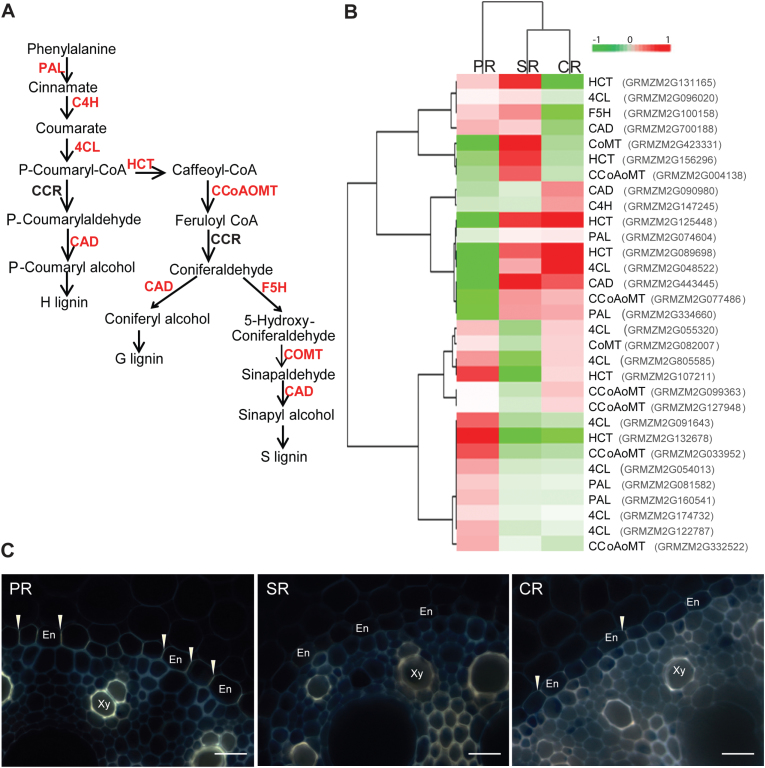
Dynamics of lignin biosynthesis among the three root types. (A) The lignin biosynthesis pathway based on MapMan. PHENYLALANINE AMMONIA LYASES (PAL), CINNAMATE-4-HYDROXYLASE (C4H), 4-COUMAROYL-COA SYNTHASE (4CL), HYDROXYCINNAMOYL TRANSFERASE (HCT), CAFFEOYL COA *O*-METHYLTRANSFERASE (CCoAOMT), CINNAMOYL-COA REDUCTASE (CCR), FERULATE 5-HYDROXYLASE (F5H), CAFFEIC ACID/5-HYDROXYFERULIC ACID 3/5-*O*-METHYLTRANSFERASE (COMT), CINNAMYL-ALCOHOL DEHYDROGENASE (CAD). Differentially expressed genes are highlighted in red. (B) Hierarchical clustering analysis of 31 differentially expressed genes predicted to be involved in lignin biosynthesis. Differential expression of genes is indicated as log_2_ of the FPKM value. (C) Cell wall lignification of transverse sections in the proximal parts of the three root types of 20mm length detected by berberine–aniline blue staining. Scale bars=25 μm; Xy, xylem; En, endodermis; PR, primary roots; SR, seminal roots; C, crown roots.

Cell proliferation and enlargement accompanied by active cell wall reorganization are the main processes that drive root growth during early development. Lignin is an important component of plant cell walls. To explore the developmental status of cell walls in the three root types, we focused on the expression of genes encoding members of the eight major families involved in lignin biosynthesis (Fig. 5A). Based on MapMan annotations, 53 genes involved in lignin biosynthesis were identified in this study, and 31 displayed differential expression across the three root types (Fig. 5B; Supplementary Table S7 at *JXB* online). These 31 genes represented seven of eight major gene families involved in lignin biosynthesis (Fig. 5A, B). Hierarchical clustering classified genes active in primary roots into a distinct group including 12 genes with higher expression in this root type (Fig. 5B). This suggested that a different set of lignin biosynthesis genes was active in primary roots compared with seminal and crown roots. Moreover, expression diversification among homologs of genes encoding enzymes such as phenylalanine ammonia lyase (PAL), 4-coumaroyl-CoA synthase (4CL), cinnamyl-alcohol dehydrogenase (CAD), and caffeoyl CoA *O*-methyltransferase (CCoAOMT) was observed in the three root types (Fig. 5B). For example, two of four genes encoding PAL which control the first step of lignin biosynthesis showed significantly higher expression in primary roots, while the other two genes were preferentially expressed in seminal and crown roots (Supplementary Table S7). Only one gene (GRMZM2G068917) encoding cinnamoyl-CoA reductase (CCR) was identified in this study which was constitutively expressed in all three root types.

Subsequently, cell wall lignification was visualized in transverse sections of the three root types through berberine–aniline blue staining (see the Materials and methods). A strong staining intensity of the endodermal Casparian strip and lignified cell walls of xylem vessels was detected in the primary root, while only a very weak staining of the Casparian strip was observed in crown roots. In seminal root sections, the Casparian strip was not detected by staining. Staining intensities of cell walls of xylem vessels in crown and seminal roots were similar but substantially less than in primary roots (Fig. 5C).

## Discussion

### Morphology: grass species-specific development and variability of seminal roots

The maize root stock is composed of embryonic primary and seminal roots and of post-embryonic shoot-borne roots (reviewed in Feldman, 1994; Hochholdinger *et al.*, 2004a, b). Primary and shoot-borne roots are formed in all cereal species, while seminal roots are present in maize (Robertson *et al.*, 1979), wheat (Erdelska and Vidovencova, 1993), and barley (Luxová, 1986), but not in rice (Morita and Abe, 1994), sorghum (Singh *et al.*, 2010), and the grass model species brachypodium (Watt *et al.*, 2009). Remarkably, the formation of seminal roots is not related to the phylogenetic relationship of these species. For instance, despite their close phylogenetic relationship (Schnable and Freeling, 2011), maize does form seminal roots while sorghum does not. Seminal roots might have therefore evolved independently in these species as functional adaptation to changing environmental challenges.

The number of seminal roots in modern maize is highly variable within a genotype and between different genotypes. While the emergence of seminal roots in the course of grass evolution remains obscure, an increase of seminal root number has been observed during the domestication of maize, barley, and wheat. Modern maize (*Zea mays* ssp. *mays*) was domesticated ~9000 years ago from its ancestor teosinte (*Z. mays* ssp. *Parviglumis*; Matsuoka *et al.*, 2002; Piperno *et al.*, 2009) and has since then been subjected to intensive improvement from maize landraces (Hufford *et al.*, 2012). A study by Burton *et al.* (2013) revealed that the majority of teosinte accessions (62%) did not form seminal roots, while the remaining accessions only formed a maximum of three seminal roots. In contrast, maize landraces formed on average 3.9 seminal roots, ranging between one and 11 per plant (Burton *et al.*, 2013). This is in the range of the 30 modern maize inbred lines that were surveyed in this study. They formed on average 3.7 seminal roots and between 0 and 10 per plant (Supplementary Table S1 at *JXB* online). Consistently, barley landraces and their modern cultivars formed significantly more seminal roots than their ancestors (Grando and Ceccarelli, 1995). Similarly, increased numbers of seminal roots were observed in domesticated wheat genotypes (Robertson *et al.*, 1979). It was suggested that root traits were probably inadvertently selected for during domestication because of their crucial role in anchorage and soil resource uptake (Lynch and Brown, 2006). An increased number of seminal roots is probably a trait that underwent selection. The notion that maize plants would benefit from an increased number of seminal roots is supported by the observation that the number of seminal roots in maize correlates with drought tolerance at the seedling stage (Qayyum *et al.*, 2012). Similarly, it was demonstrated that the number and length of seminal roots were positively yet weakly correlated with shoot biomass in the field under low phosphorus (Zhu *et al.*, 2006). Taken together, these results imply that inadvertent selection of genotypes with increased seminal root number during maize domestication probably has contributed to the agronomic success of modern maize.

### Anatomy: cost-efficient anatomical structure of seminal roots in the maize inbred line B73

The early establishment of the maize seedling root system depends on the availability of carbohydrates stored in the starchy endosperm of the seed (Lopes and Larkins, 1993). These carbohydrates provide the primary energy for the emergence of embryonic primary roots from the basal pole of the seed and seminal roots from the scutellar node in the first days after germination (Hochholdinger *et al.*, 2004a). Nutrient acquisition depends more on the root surface than on root volume. Formation of a single primary root and several seminal roots might be an efficient way of allocating seed-stored carbohydrates for maize seedling root system establishment and thus enable an efficient acquisition of soil resources and early anchorage of the seedling.

Quantification of anatomical traits in the inbred line B73 revealed thinner seminal roots with significantly fewer cortical cell files relative to primary and crown roots (Fig. 1C). A reduced number of cortical cell files was associated with increased drought tolerance in maize (Chimungu *et al.*, 2014). Moreover, the ratio of meta-xylem vessels to the whole stele area was significantly increased in B73 seminal roots compared with the primary root, while the ratio of stele to transverse area showed no difference (Fig. 1C). The ratio of the stele to transverse area probably influences the root system cost and function (Burton *et al.*, 2013). Meta-xylem vessels in the stele are the largest vascular elements and mainly responsible for water and nutrient transport from the root to the shoot (Hochholdinger, 2009). A larger stele is likely to accommodate more xylem vessels for increased water and nutrient transport, whereas initial carbon investment for establishing a larger stele is greater on a per root basis (Burton *et al.*, 2013). The observation that seminal roots of the modern maize inbred line B73 displayed a greater ratio of meta-xylem vessels to the whole stele area but a similar ratio of stele to transverse area compared with primary root suggests they might be better equipped for efficient water and nutrient transport without additional costs for stele establishment.

### Comparative transcriptome analyses highlight the uniqueness of seminal roots

RNA-seq of different root types of the maize reference inbred line B73 revealed a distinct transcriptomic landscape of seminal versus primary and crown roots. First, hierarchical clustering and PCA of all expressed genes revealed that seminal roots were only distantly related to primary and crown roots which clustered closely together (Fig. 2A, B). This correlates with the unique anatomy of seminal roots of the inbred line B73 that is distinct from the more similar primary and crown roots (Fig. 1B, C). Secondly, substantially more genes were exclusively expressed in seminal roots than in the other root types (Fig. 2C). Moreover, more genes were differentially expressed in seminal roots and primary and crown roots than between primary and crown roots (Fig. 2D; Supplementary S2B at *JXB* online). Similarly, observations that anatomical or physiological differences correlate with transcription profiles have been made in maize (Sekhon *et al.*, 2011; Downs *et al.*, 2013), rice (Wang *et al.*, 2010), Arabidopsis (Ma *et al.*, 2005), and tobacco (Edwards *et al.*, 2010). This suggests that tissue or organ identity is a primary factor that explains transcriptome variation (Downs *et al.*, 2013; Raherison *et al.*, 2015), while changing environmental conditions affect a much smaller set of genes, as demonstrated for salt stress in maize (Zhang *et al.*, 2015) and a plethora of stresses in Arabidopsis (Aceituno *et al.*, 2008). Functional annotation of differentially expressed genes further revealed root type-specific developmental functions (Fig. 3B). Remarkably, seminal roots displayed distinct enriched functional categories compared with primary and crown roots which were relatively similar (Fig. 3B), suggesting functional divergence of seminal roots. Some of these differences will be discussed in the following sections.

### Distinct roles of transcriptional regulators in seminal roots

Hierarchical clustering of transcriptional regulators suggested specific transcriptional regulation in seminal roots compared with similar regulation in primary and crown roots (Fig. 4A). For instance, members of the ARF and HD-ZIP families were enriched in primary and crown roots (Fig. 4B). Both families are involved in growth, development, and cell division, and respond to environmental stimuli (Van Ha *et al.*, 2013; Chen *et al.*, 2014). In contrast, members of the LBD, G2-like, bHLH, ERF, DOF, and WRKY families were over-represented in seminal roots (Fig. 4B). Among those, the LBD protein RTCS of maize is instrumental in seminal root initiation (Taramino *et al.*, 2007). Moreover, G2-like, bHLH, ERF, DOF, and WRKY TFs play different roles in root growth and development including xylem or phloem cell differentiation, cell elongation, root hair development, stress responses, ethylene responses, and ABA signaling (Zhao *et al.*, 2005; Nakano *et al.*, 2006; Feller *et al.*, 2011; Rushton *et al.*, 2012; Guo and Qiu, 2013). Coincidently, genes involved in ABA metabolism including many ABA-induced genes were significantly enriched in seminal roots (Fig. 3B). ABA is produced under osmotic stress conditions such as drought and salinity, and plays an important role in stress response and tolerance of plants (Jakab *et al.*, 2005). Specific enrichment of members of these TF families related to stress response in seminal roots suggests that stress activates different signaling pathways in seminal roots of the inbred line B73.

### Diversification of cell wall lignification among the root types

The secondary metabolite lignin is an important component in secondary cell walls of developing maize roots (Zeier *et al.*, 1999). Lignin is deposited in the secondary cell walls of endodermis cells and xylem vessels to strengthen the root and enhance plant anchorage (Vermerris *et al.*, 2010). Compared with Arabidopsis, the number of lignin biosynthesis genes has substantially increased in maize (Penning *et al.*, 2009). Dynamic expression patterns of lignin biosynthesis genes across the three root types underpin the diversity and complexity of transcriptional regulation in maize root development (Fig. 5A, B). Remarkably, while seminal roots displayed distinct features for most anatomical and transcriptomic features from primary and crown roots, hierarchical clustering revealed similar expression patterns for lignin biosynthesis genes in seminal and crown roots which were distinct from expression profiles in primary roots. Organ-specific expression patterns of lignin genes underscoring the diversification of the lignin pathway in maize have also been observed for other vegetative organs (Sekhon *et al.*, 2011). Histochemical staining further supported the differences observed in the transcriptomic profiles by revealing differences in cell wall lignification of the endodermis and xylem vessels between the three root types (Fig. 5C). Increased cell wall lignification coupled with diversification of the lignin pathway in primary roots might indicate relatively active formation and regulation of secondary cell walls in primary root development (Fig. 5B, C). Similarly, the *brown midrib2* (*bm2*) mutant of maize which is defective in cell wall biosynthesis displayed tissue-specific patterns of lignification (Vermerris and Boon, 2001). Excessive lignin deposition was observed in the mutant *rum1* of maize which is defective in vasculature formation (Zhang *et al.*, 2014).

### Root type-specific expression of genes of the auxin signal transduction pathway

Genes involved in auxin signal transduction are important for various aspects of maize root development. The *Aux/IAA* gene *rum1* (Woll *et al.*, 2005) controls seminal and lateral root initiation in primary roots and depends on the activity of ZmARF34 (von Behrens *et al.*, 2011). This is in line with preferential expression of *ZmARF34* in primary roots in the present study. Interaction of RUM1 with ZmIAA33 has been revealed by Ludwig *et al.* (2014). The *ZmIAA33* gene that emerged after the whole-genome duplication of maize (Ludwig *et al.*, 2014) displayed peak expression in seminal roots in this study. Consistently, *ZmIAA33* displayed substantially higher expression in seminal roots relative to primary and crown roots (Ludwig *et al.*, 2014). Sorghum (Singh *et al.*, 2010) and rice (Morita and Abe, 1994), which both do not form seminal roots, do not have homologs of *ZmIAA33* (Ludwig *et al.*, 2014). These findings support functions of auxin-related genes with root type-specific expression profiles in maize root development.

In summary, seminal roots of the maize inbred line B73 display a distinct anatomical structure and transcriptomic landscape compared with primary and crown roots related to the different functions of these root types during development. The formation of seminal roots in modern maize might have allowed maize to explore new habitats which were inaccessible for maize progenitors without seminal roots.

## Supplementary Data

Supplementary data are available at *JXB* online.

Figure S1. Serial transverse sections of 30, 60, and 90mm long primary (PR), seminal (SR), and crown roots (CR) of maize in 10mm increments.

Figure S2. MA and Volcano plots displaying the correlation of gene expression changes versus mean expression in the pairwise comparisons.

Figure S3. Singular enrichment analyses (SEA) with AgriGO revealed significantly enriched GO terms for differentially expressed genes in the three pairwise comparisons.

Table S1. Summary of seminal root counts of 30 modern maize inbred lines.

Table S2. RNA-seq output and mapping results.

Table S3. List of 24 687 genes expressed in at least one of the three root types in four biological replicates.

Table S4. Classification of the 12 207 differentially expressed genes among the three root types into 12 K-means clusters.

Table S5. List of 202 classical maize genes differentially expressed in the three root types.

Table S6. List of 1252 differentially expressed genes involved in transcriptional regulation.

Table S7. List of 31 differentially expressed genes involved in lignin biosynthesis.

Supplementary Data
