# Supplementary material for: Transcriptomic and anatomical complexity of primary, seminal, and crown roots highlight root type-specific functional diversity in maize (Zea mays L.)
Source: J Exp Bot. 2015 Nov 30;67(4):1123–35. doi: 10.1093/jxb/erv513 (PMC4753849; doi:10.1093/jxb/erv513)
Supplement: Supplementary Data [file supp_67_4_1123__index.html]

Transcriptomic and anatomical complexity of primary, seminal, and crown roots highlight root type-specific functional diversity in maize (Zea mays L.) — Transcriptomic and anatomical complexity of primary, seminal, and crown roots highlight root type-specific functional diversity in maize (Zea mays L.) — Transcriptomic and anatomical complexity of primary, seminal, and crown roots highlight root type-specific functional diversity in maize (Zea mays L.) — Supplementary Data 

# Transcriptomic and anatomical complexity of primary, seminal, and crown roots highlight root type-specific functional diversity in maize (*Zea mays* L.)

## Supplementary Data

Data files

- Supplementary\_fig.\_S1\_S3.pdf - Supplementary Data
- Supplementary\_Tables\_S1.xlsx - Supplementary Data
- Supplementary\_Tables\_S2.xlsx - Supplementary Data
- Supplementary\_Tables\_S3.xlsx - Supplementary Data
- Supplementary\_Tables\_S4.xlsx - Supplementary Data
- Supplementary\_Tables\_S5.xlsx - Supplementary Data
- Supplementary\_Tables\_S6.xlsx - Supplementary Data
- Supplementary\_Tables\_S7.xlsx - Supplementary Data
