# Supplementary material for: Transcriptomic and anatomical complexity of primary, seminal, and crown roots highlight root type-specific functional diversity in maize (Zea mays L.)
Source: J Exp Bot. 2015 Nov 30;67(4):1123–35. doi: 10.1093/jxb/erv513 (PMC4753849; doi:10.1093/jxb/erv513)

Fig. S1 Serial transverse sections of 30, 60 and 90 mm primary (PR), seminal (SR) and crown roots (CR) of maize in 10 mm increments. Scale bars: 200 µm.

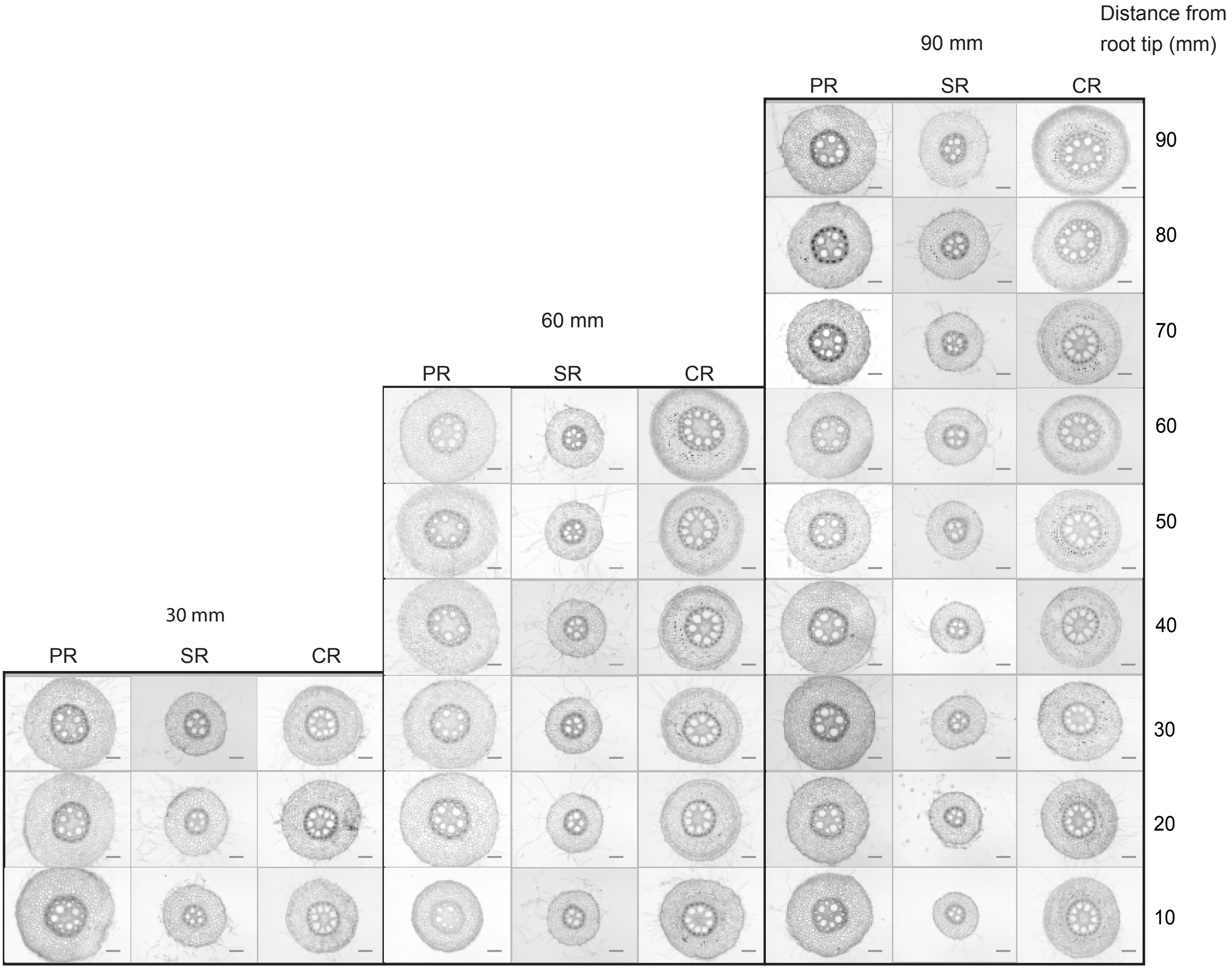

Fig. S**2** (a) Overview of the correlation of gene expression changes versus expression intensity by plotting $\log_2$ fold-changes of genes against their mean expression in pairwise comparisons. (b) Volcano plots showing differentially expressed genes in the three pairwise comparisons. Numbers of differentially expressed genes (FDR <5%) are indicated. Genes with FDR <5%, and $|\log_2 Fc| \geq 1$ are given in brackets. PR: primary roots; SR: seminal roots; CR: crown roots.

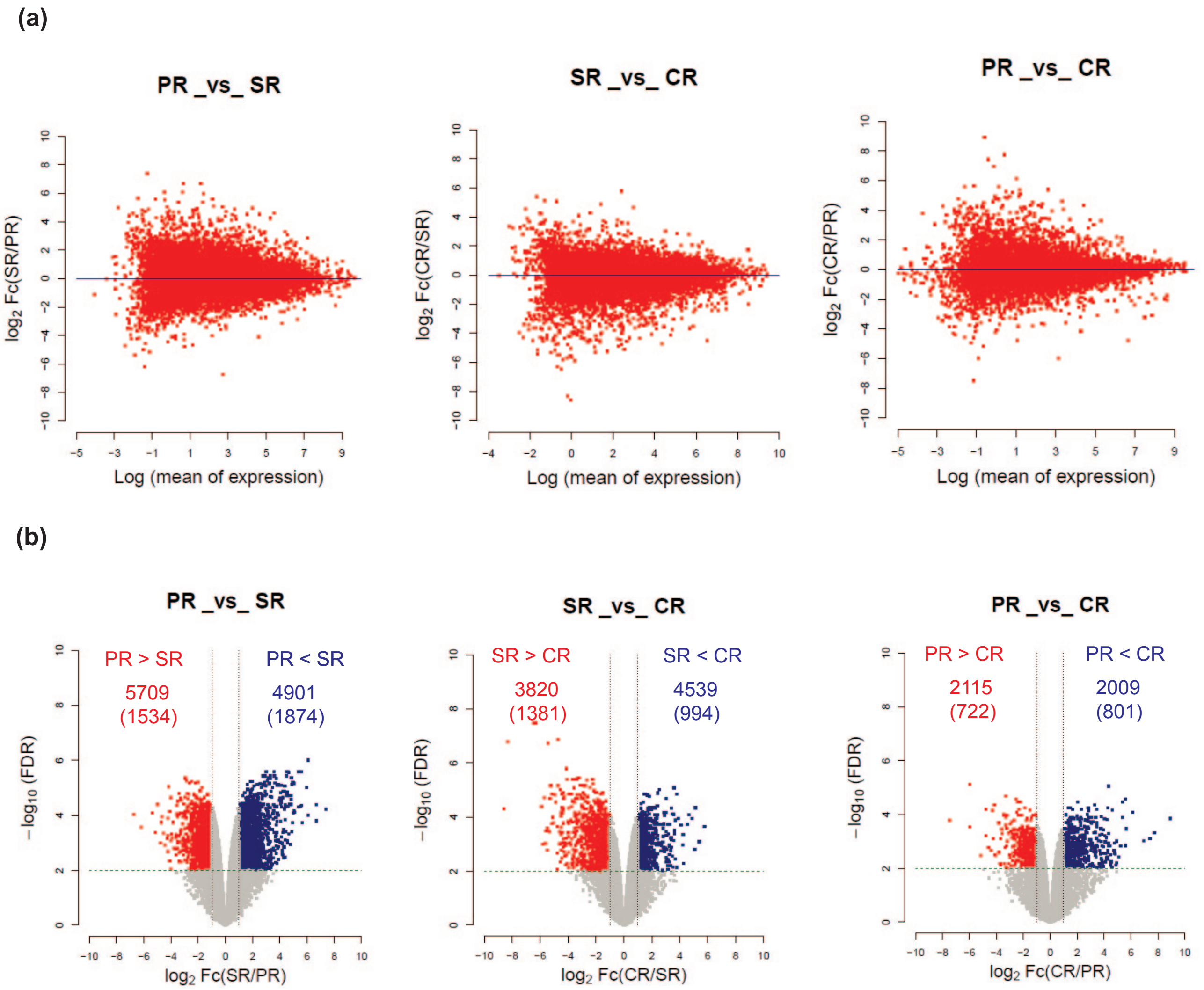

Fig. S3 Singular enrichment analyses (SEA) with AgriGO revealed significantly enriched GO terms (indicated by asterisks) for differentially expressed genes in the three pairwise comparisons (FDR <5%, $|\log_2 Fc| \geq 1$). The input list were the differentially expressed genes in each comparison. The 24,687 expressed genes in this study were used as reference. PR: primary roots; SR: seminal roots; CR: crown roots.

(a) PR *vs* SR

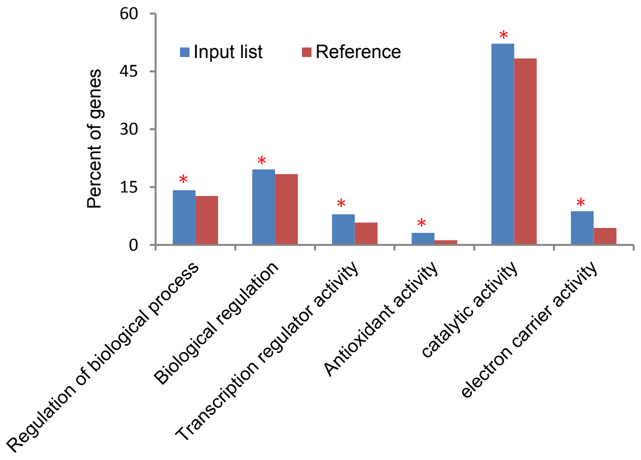

(b) PR *vs* CR

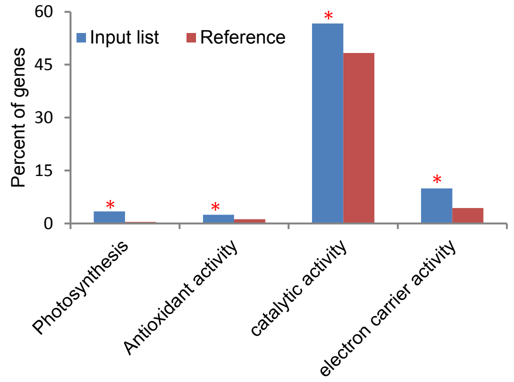

(C) SR vs CR

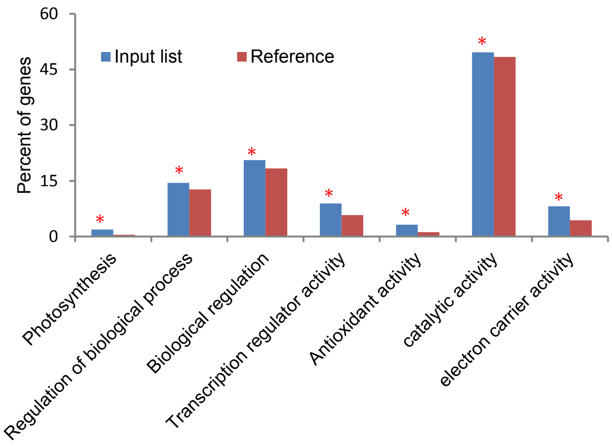

Supplement: Supplementary Data [file supp_erv513_Supplementary_fig._S1_S3.pdf]
